# Palliative Care Involvement in Hospitalized Amyotrophic Lateral Sclerosis Patients

**DOI:** 10.3390/ijerph23081081

**Published:** 2026-08-19

**Authors:** Sumeet Bhardwaj, Anita Chakraborty, Jillian Mead, Kalli Stilos

**Affiliations:** 1 Division of Palliative Medicine, Department of Family and Community Medicine, Sunnybrook Health Sciences Centre, Toronto, ON M4N 3M5, Canada; 2 Division of Palliative Care, University of Toronto, Toronto, ON M5S 1A1, Canada; 3 Lawrence Bloomberg Nursing Program, University of Toronto, Toronto, ON M5S 1A1, Canada

**Keywords:** Amyotrophic Lateral Sclerosis, palliative care consult service, hospitalized patients, symptom management, multidisciplinary care

## Abstract

**Highlights:**

**Public health relevance—How does this work relate to a public health issue?**

- Amyotrophic lateral sclerosis (ALS) is a life-limiting disease with substantial physical, psychosocial, and caregiver burden requiring coordinated multidisciplinary care.
- Delayed access to specialist palliative care may contribute to unmet supportive care needs, crisis hospitalizations, and inequities in end-of-life care for people with ALS.

**Public health significance—Why is this work of significance to public health?**

- This study identifies patterns of very late specialist palliative care referral among hospitalized ALS patients, highlighting opportunities to improve care delivery across the disease trajectory.
- The findings support earlier integration of palliative care within multidisciplinary ALS services to promote advance care planning, symptom management, and goal-concordant care.

**Public health implications—What are the key implications or messages for practitioners, policy makers and/or researchers in public health?**

- Health systems should integrate specialist palliative care earlier in multidisciplinary ALS care pathways and strengthen processes that support timely referral and advance care planning.
- Future research should evaluate whether early integrated neuropalliative care improves patient outcomes, reduces acute care utilization, and aligns care more closely with patient preferences.

**Abstract:**

Amyotrophic lateral sclerosis (ALS) is a progressive neurodegenerative disease associated with significant physical, psychological, and social distress. Given its terminal nature and high symptom burden, early integration of palliative care is essential. The inpatient Palliative Care Consult Team (PCCT) provides specialist palliative care to hospitalized patients with ALS. Hospitalizations are common throughout the ALS disease trajectory, particularly as patients experience progressive functional decline, respiratory compromise, and increasing care needs, making inpatient encounters important opportunities for specialist palliative care involvement. To extend palliative care beyond the inpatient setting and facilitate earlier involvement, an ALS ambulatory clinic was established in 2018. Despite the importance of palliative care, limited data describes its involvement among hospitalized ALS patients. This retrospective review examines the relationship between the PCCT and ALS patients admitted to a tertiary care facility between 2006–2019. Data collected included patient demographics, referral indications, clinical course, and disposition. Most patients referred to the PCCT had poor functional status and a guarded prognosis at initial consultation. Symptom management and support for complex decision-making were leading reasons for referral. Approximately half of patients died in the hospital within three months of referral. Most deaths occurred within one week, underscoring the importance of timely palliative care.

## 1. Introduction

Amyotrophic Lateral Sclerosis (ALS) is a progressive neurodegenerative disease that manifests in a wide range of distressing physical and psychosocial symptoms. Physically, patients experience weakness, spasticity, dysarthria, dysphagia, dyspnea, and pseudobulbar affect [1,2]. In addition to the physical burden, ALS is associated with significant psychosocial challenges, including depression, anxiety, social isolation, loss of independence, and existential distress [3,4,5]. These symptoms impact not only patients, but also caregivers, who often experience emotional, physical, and financial strain. The complex and multifaceted nature of ALS necessitates a multidisciplinary approach to care throughout all phases of the illness to address the diverse domains of suffering and distress [6].

Palliative care is a specialized approach to care that focuses on improving quality of life for patients and families affected by serious illness through symptom management, psychosocial support, and assistance with complex decision-making and goals-of-care discussions [7]. Given the terminal nature of ALS and the substantial burden it places on patients and their families, the involvement of specialist palliative care is essential. Specialist palliative care has been shown to improve outcomes in individuals with serious illnesses by addressing symptom management, providing psychosocial support, and assisting in advance care planning conversations [1]. Furthermore, data suggest that both patients and caregivers desire the involvement of palliative care specialists in their care journey [8]. However, despite these potential benefits, referral to palliative care in ALS may occur late in the disease trajectory due to prognostic uncertainty, evolving care needs, and challenges recognizing the appropriate timing for specialist involvement [9,10]. Beyond symptom optimization, there is growing evidence that palliative care engagement in the ALS population promotes timely and meaningful goals-of-care discussions in both inpatient and outpatient settings [9,10].

Evidence from ALS literature supports this shift in practice. Multidisciplinary models of care that integrate palliative care have been shown to improve quality of life and mental well-being for patients with ALS [11] and are associated with improved survival compared to standard neurological care. Broader reviews further demonstrate that coordinated multidisciplinary and palliative approaches can reduce hospitalizations, improve patient satisfaction, and decrease mortality by addressing the complex physical and psychosocial needs of ALS patients [12]. Palliative care is now recognized as an essential component of ALS care across the disease trajectory, supporting symptom management, advance care planning, and end-of-life care [13].

Complex decision-making around medical interventions such as the insertion of a percutaneous endoscopic gastrostomy (PEG) tube or a tracheostomy can be particularly challenging to patients with ALS. These decisions require careful navigation of risks, benefits, and alignment of care plans with patients’ individual values and goals. Goals-of-care conversations should ideally be guided by skilled clinicians with expertise in palliative care. While the role of palliative care in oncology is well studied, there remains a gap in understanding its specific impact on patients with ALS. Some studies have demonstrated an improvement in quality of life and symptom management with specialist palliative care in the ALS population [10]. This descriptive paper aims to explore and better define the relationship between specialist-level palliative care and patients diagnosed with ALS referred to an inpatient palliative care consult service.

### Setting

Sunnybrook Health Sciences Centre (SHSC) in Toronto, Ontario, Canada, is a large tertiary care hospital that operates the largest ALS clinic in Canada and one of the largest globally. As of 2026, the clinic follows approximately 250 patients and serves as the sole specialized ALS clinic for the Greater Toronto Area and surrounding regions. Affiliated with the University of Toronto Neurosciences Program, the clinic provides comprehensive multidisciplinary care through a team of neurologists, respirologists (pulmonologists), nurses, social workers, occupational therapists, registered dietitians, respiratory therapists, and speech-language pathologists, with routine referrals to physiatrists and psychiatrists.

Palliative care physicians formally joined the ALS clinic in 2018, expanding the scope of holistic care available to patients and families. This initiative followed an earlier departmental effort that relied on ad hoc palliative care consultation, which did not achieve consistent integration into routine ALS care. The transition to a dedicated embedded model was intended to improve continuity, accessibility, and collaboration between palliative care and the ALS multidisciplinary team. The service has since expanded and is now provided by three palliative care physicians, who divide ALS clinic responsibilities alongside their broader clinical practices. This model was further supported by growing evidence demonstrating that early integration of palliative care within multidisciplinary ALS clinics improves symptom management, enhances emotional well-being, strengthens support for patients and caregivers, facilitates advance care planning, and promotes goal-concordant care while reducing unnecessary hospitalizations, burdensome interventions, and potentially lowering healthcare costs [12,14,15,16].

Within SHSC, the inpatient Palliative Care Consult Team (PCCT) is an interprofessional group consisting of advanced practice nurses, medical trainees, and staff physicians. PCCT provides specialist palliative care consultations for hospitalized patients, focusing on managing complex symptoms and facilitating goals-of-care discussions with patients and their families during particularly challenging and distressing periods.

Referrals to PCCT come from a diverse range of hospital departments, including oncology, internal medicine, surgery, nephrology, cardiology, and trauma, among others. As evidence has increasingly demonstrated the benefits of specialist palliative care involvement in serious illnesses, including improved symptom management, better alignment of care with patient goals, and enhanced communication and decision-making, referrals for patients with ALS to the PCCT have correspondingly risen. This trend reflects growing recognition within the broader healthcare system of the important role of palliative care in addressing the complex needs of this population.

## 2. Materials and Methods

### Methods

For every patient referred to and seen by the PCCT, a standardized database sheet is completed by the consultant which captures integral demographic and clinical information. This retrospective study examined data collected on ALS patients seen by the PCCT from January 2006 to March 2019. This time frame was selected as it corresponds to a period during which dedicated departmental funding supported comprehensive and consistent data collection. This funding has since ended, limiting the availability of more recent standardized data. Reviewing this cohort provides an opportunity to characterize patterns of palliative care involvement and patient outcomes in ALS prior to formal integration of the PCCT into the care delivery pathway, generating insights that may inform current clinical practice and future service planning. Extracted data included demographic and clinical characteristics including gender, age, primary diagnosis, Palliative Performance Scale (PPS) score, reason for referral, time from hospital admission to palliative care consultation, time from consultation to disposition or death, disposition location, and code status.

PPS is a validated functional assessment tool used in palliative care to describe a patient’s functional status based on mobility, activity level, self-care, oral intake, and level of consciousness. PPS was collected as an indicator of overall functional decline and disease burden at the time of palliative care consultation, as these factors may inform clinical assessment, prognosis, and care planning.

Code status, including documentation of do-not-resuscitate (DNR) status, was collected as an indicator of goals-of-care decision-making and advance care planning. Given the progressive and terminal nature of ALS, decisions regarding life-sustaining interventions require careful consideration of patient preferences, values, and evolving disease-related needs.

This study was reviewed and approved by the SHSC Research Ethics Board (study ID: 6863). Waiver for consent for research participants was approved as all participants had died by the time of data collection, and it would have been impracticable to contact all bereaved caregivers to obtain consent.

## 3. Results

A total of 88 patients with ALS were seen by the PCCT at SHSC over the 13-year period from January 2006 to March 2019. On average, the PCCT sees approximately 875 inpatients per year [17]. Of the 88 patients with ALS seen by the PCCT, 85 were seen as inpatients and 3 were seen as outpatients. The cohort consisted of 48 women (55%) and 40 men (45%). The small number of outpatients reflected the ALS clinic being in its infancy. Most of the patients (75%) were over the age of 65 at the time of consultation (Table 1). Code status at initial consultation was available for 85 patients; of these, 73 (86%) had a documented Do Not Resuscitate (DNR) order and 12 (14%) were designated Full Code.

The most common reasons for referral to PCCT were “advance care planning and discharge planning” (41%) and “pain and symptom management” (35%). At the time of initial consultation, 97% of patients had a PPS score of 40% or less, indicating a high level of functional impairment. Twenty-three percent were functioning at a PPS of 10%, reflecting end-of-life care needs.

**Figure 1:**
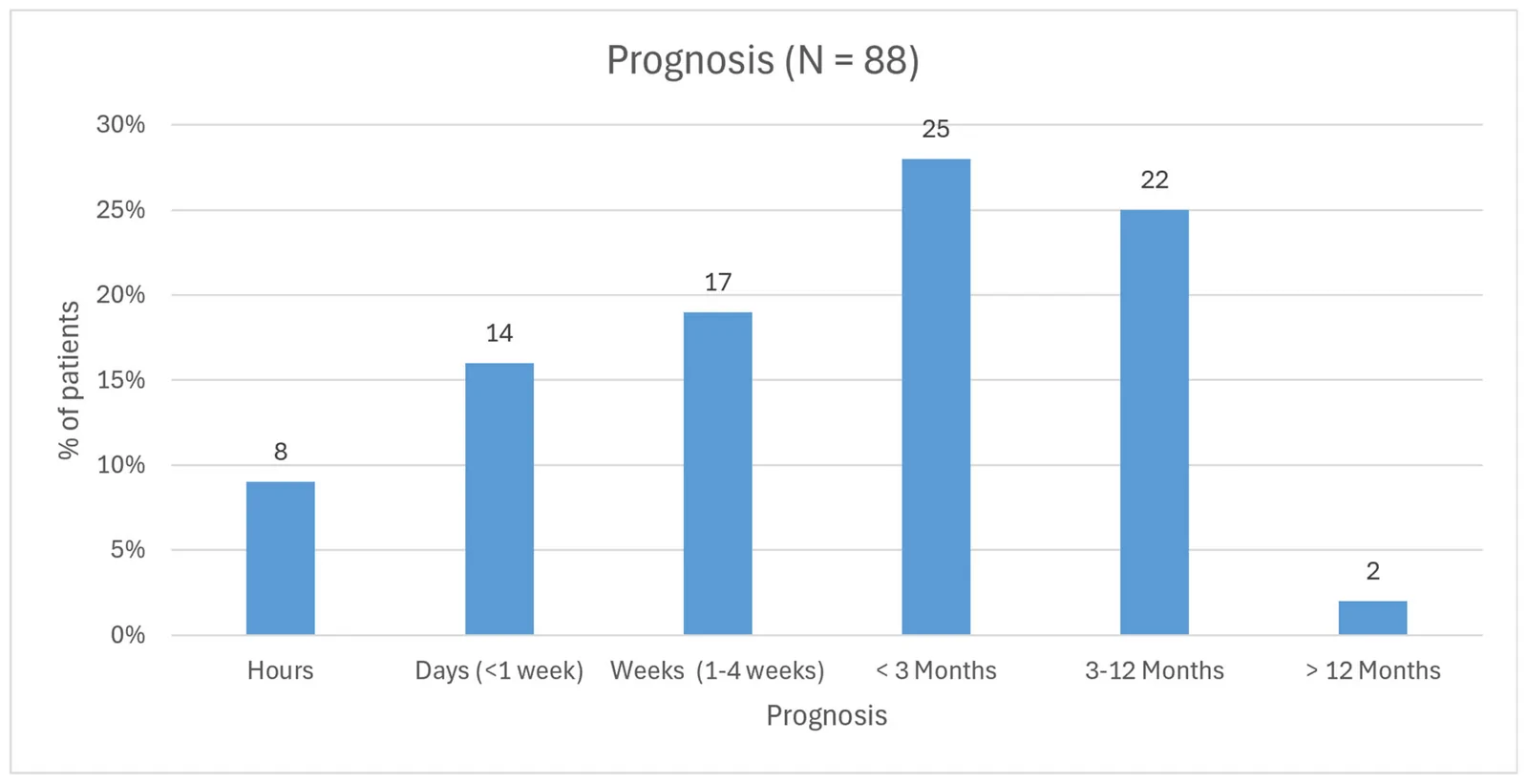
Estimated Prognosis by Palliative Care Specialist at Time of Consultation.

Twenty-five percent of the 88 patients referred had an estimated survival of less than one week at the time of initial consultation (Figure 1). However, of the 42 patients who died in the hospital, 74% of them died within one week following initial consultation, while none of them survived beyond three months (Table 2).

**Figure 2:**
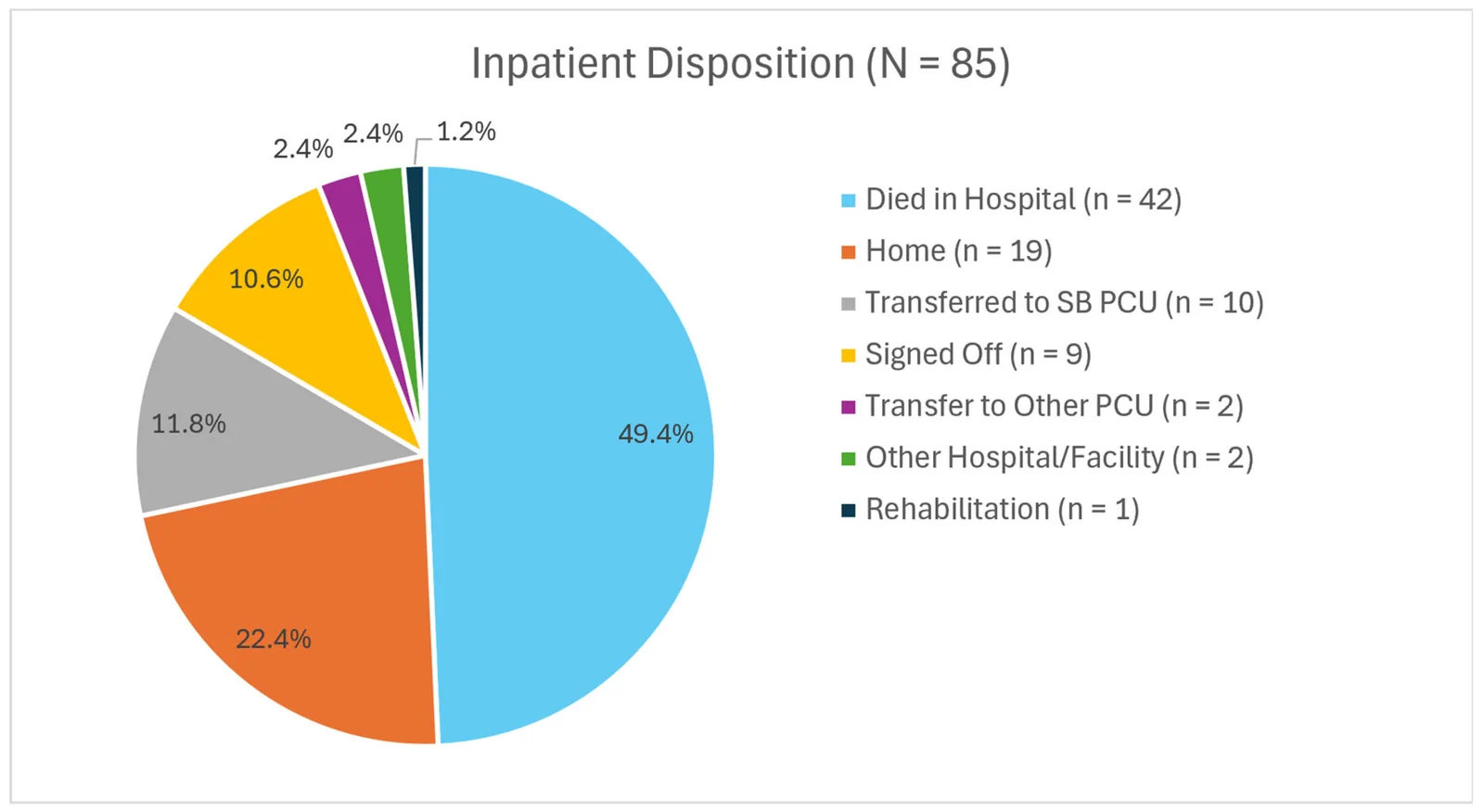
Disposition of Inpatient ALS Patients Consulted on. Three patients, who were seen in the outpatient clinic, were not included in this figure due to the small size of this subgroup. SB PCU = Sunnybrook Palliative Care Unit; PCU = Palliative Care Unit.

Forty-nine percent of ALS patients died in acute care settings, while only 12% were transferred to SHSC’s Palliative Care Unit (PCU). Additionally, 22% of patients were discharged home following their hospital stay (Figure 2).

## 4. Discussion

### 4.1. Reason for Referral: Advance Care Planning and Symptom Management

This descriptive study adds valuable insights into the growing body of literature on ALS patients referred to an inpatient specialist palliative care team. Our findings reveal that the most common reason for referral to the Palliative Care Consult Team (PCCT) was for “advance care planning and discharge planning”. This is consistent with the disease’s progressive and irreversible nature, with a median survival of 2 to 4 years, requiring prompt and informed decision-making [14,18]. Early initiation of advance care planning is increasingly recognized as an essential component of ALS care. Structured advance care planning (ACP) discussions early in the disease trajectory can facilitate meaningful conversations about preferences for future interventions, thereby supporting patient autonomy and preparedness for disease progression [19].

These findings are further supported by a recent 15-year program review conducted by our team [17], which demonstrated that approximately 30% of all new palliative care referrals between 2006 and 2021 included requests related to advance care planning or goals-of-care discussions. These findings may reflect increasing recognition of the benefits of early palliative care involvement in supporting advance care planning, goals-of-care discussions, and patient-centred decision-making in serious illness care [20].

Additionally, the complexity and relatively low prevalence of ALS may create challenges in coordinating the wide range of community, home care, and supportive services required throughout the disease trajectory. Palliative care teams play a critical role not only in facilitating advance care planning, but also in supporting discharge planning, connecting patients and families with available resources, and helping them navigate an increasingly complex healthcare system.

Requests for symptom management were the second most common reason for referral to our service. Patients with ALS often experience distressing symptoms such as pain, weakness, muscle cramps and degenerations, dysarthria, dysphagia, dyspnea along with psychosocial issues such as fear, anxiety and impaired cognitive ability [21]. Despite the substantial physical and psychosocial burden associated with ALS, referral to palliative care often occurs late. This delay may be related to uncertainty regarding prognosis, evolving care priorities, and misconceptions among patients, families, and healthcare providers that palliative care is primarily associated with end-of-life care rather than supportive care throughout the disease trajectory [22]. Brizzi et al.’s [22] initial study of specialist palliative care involvement in a multidisciplinary ALS clinic highlighted that patients and caregivers often prioritize advanced care planning and guidance on navigating the journey of the disease rather than immediate symptom relief alone. Their findings emphasize the importance of longitudinal conversations and early integration of palliative care within ALS clinics. This is particularly relevant to our study, where advance care planning was the most common reason for referral, but occurred late in the disease course, suggesting a missed opportunity for earlier engagement. While this review identifies essential information needs at end-of-life, it does not fully capture the variability in communication preferences among patients and families.

### 4.2. Timing of Referral and Late Palliative Care Involvement

At our institution, 74% of ALS patients died within one week of palliative care consultation, suggesting that palliative care was integrated only in the very late stages of the disease course. This pattern of delayed referral raises serious concerns about the ability to deliver meaningful, patient-centered care, and is likely associated with increased distress for both patients and their families [23]. In the broader palliative care literature, several standardized screening tools have been developed to help clinicians identify patients with advanced chronic conditions who may benefit from specialist palliative care earlier in the disease course. Tools such as the Supportive and Palliative Care Indicators Tool (SPICT) and the Necesidades Paliativas (NECPAL) tool incorporate general indicators of deterioration (e.g., functional decline, frequent hospital admissions) and disease specific criteria to flag patients with progressive non-cancer illnesses who may have unmet palliative care needs and warrant formal assessment and referral [24,25]. While no tool is perfectly predictive, these instruments offer structured approaches to prompt clinicians to consider palliative care involvement before patients and families present in crisis. Tools may support more proactive and systematic referral practices in populations with variable disease trajectories such as ALS [25].

Notably, 86% of patients already had DNR status established at the time of initial PCCT consultation, further suggesting that many goals-of-care decisions were made prior to specialist palliative care involvement. While this may reflect prior discussions with primary teams, our data does not capture the extent of community-based palliative care involvement that may have influenced these decisions. Conversely, the remaining 14% of patients had not yet established limitations on life-sustaining treatment, representing a subgroup for whom hospital admission and PCCT involvement may have provided a critical opportunity to explore goals of care in greater depth.

An important system-level change at SHSC was the implementation of an Advance Care Planning/Goals of Care documentation tab within the electronic medical record accessible to physicians, nurse practitioners, physician assistants, and social workers [17,26]. The tab was implemented to facilitate communication and continuity of care; however, our study did not evaluate its impact on this cohort of patients. Given the progressive nature of this disease, having a centralized and readily accessible documentation system is particularly valuable for ensuring communication across care settings as patients with ALS typically transition between outpatient, inpatient, and community-based care.

Goals-of-care discussions are grounded in the presence of a strong therapeutic relationship between patients, families, and healthcare providers. Exploring goals of care is often a complex, longitudinal process that unfolds over multiple encounters, allowing for the development of this relationship [13]. Such a relationship is essential for sound decision-making, and the formulation of plans aligned with patients’ values and preferences. Our data suggest that late PCCT involvement limits this process, potentially leaving patients and families feeling rushed, overwhelmed, and unable to fully process information to make informed decisions.

### 4.3. Prognostication Challenges in ALS

In non-cancer illnesses and conditions of frailty, clinicians, patients, and caregivers frequently fail to accurately recognize the end-of-life period [27]. Prognostic uncertainty, particularly in diseases with variable and unpredictable trajectories such as ALS, can impede or delay timely palliative care involvement and meaningful serious illness conversations [28,29]. Unlike many cancers, ALS often follows a fluctuating course with periods of relative stability followed by rapid decline, making it difficult for clinicians to identify a clear transition to end-of-life care.

Prognostic uncertainty can also limit the establishment of effective therapeutic relationships. Strong therapeutic relationships typically develop over time through repeated interactions, allowing clinicians to build trust, understand patient values, and support shared decision-making. When referrals to palliative care occur late in the disease trajectory, there is insufficient time to develop this rapport. As a result, conversations about goals of care may occur in crisis situations, where patients and families are under significant emotional distress, reducing their ability to process information and engage in goal concordant decision-making. Late palliative care involvement therefore disadvantages both patients and providers by truncating the time available to build trust and explore nuanced care preferences which can subsequently lead to poorer quality of life experienced by patients [18]. However, this raises the question of the respective roles of palliative care consultants versus primary treating teams initiating these discussions and fostering therapeutic relationships. Emerging literature suggests that high-quality serious illness communication should be a shared responsibility, with primary teams introducing and normalizing goals-of-care conversations early, and palliative care specialists providing additional expertise in complex communication and decision-making [30,31].

As has been previously demonstrated [32], our study indicates that PCCT providers generally overestimate survival, which underscores the challenges of accurate prognostication in ALS patients. The inaccuracy of prognostication may stem from its illness course. The final stage of ALS, often marked by respiratory failure, is perceived by many clinicians as distinct from the typical course of other terminal illnesses, making it more difficult to recognize and manage [33]. Moreover, while the PPS can be valuable for estimating survival time in cancer patients, additional factors should be considered when predicting survival in non-cancer patients [34].

### 4.4. Location of Death and Health System Implications

Our data reveal that nearly half (49%) of ALS patients seen by PCCT died in the acute care setting, with only 12% receiving end-of-life care in the hospital’s Palliative Care Unit (PCU). These findings are consistent with those reported by Zwicker et al. [35] who conducted a retrospective, population-based cohort study of Ontario decedents from 2013 to 2015, examining demographics, health care utilization, and costs in the last year of life of ALS patients. Their study demonstrated underutilization of inpatient palliative care units and hospice despite evidence that these services can improve end-of-life care quality and reduce health care costs.

A recent cohort study found that hospitals were the most common place of death for ALS patients (46%), exceeding home or nursing home deaths. Even among those referred to specialized palliative care, 51% still died in hospital. Importantly, only a subset of in-hospital deaths occurred in palliative care wards (~39%), meaning many died in acute care units (ICU, neurology, pulmonology) rather than dedicated PCUs [36].

The discrepancy in place of death may reflect patients’ care preferences that are not exclusively comfort-focused. At a systems level, the inability of PCUs to provide interventions such as bilevel positive airway pressure (BiPAP) may significantly limit the utility of PCU admission in the ALS cohort [37]. Studies indicate that patients and families often feel that admission to PCUs occurs too late in the disease trajectory [38], a gap that could be addressed by earlier specialist palliative care involvement. Providing BiPAP capabilities in our PCU may reduce deaths in acute care by optimizing patient comfort in appropriate settings and lowering healthcare system costs. Previous studies demonstrate that a major driver of end-of-life acute-care utilization is the lack of discharge options for patients choosing a palliative approach [39]. These constraints may be even more challenging in the setting of ALS patients, given their high symptom burden and unique care needs.

## 5. Conclusions

### 5.1. Early Integration of Palliative Care and System Improvements

Sunnybrook has taken steps to integrate palliative care earlier in the ALS care pathway. Since 2018, palliative care physicians have been embedded within the ALS ambulatory clinic, to promote earlier specialist involvement and better manage the intertwined physical, emotional, and spiritual needs of patients [15]. Evidence from the literature supports that early integration of palliative care into multidisciplinary ALS clinics improves symptom management, enhances emotional well-being, and provides stronger support for both patients and caregivers [14]. Furthermore, early palliative care may help avoid unnecessary or unwanted hospital admissions and aggressive medical interventions, thereby leading to more goal-concordant care and potentially reducing healthcare costs [16].

Improving the timeliness of palliative care consultation is beneficial not only for patients and families but also for healthcare systems, with evidence demonstrating improvements in quality of life, symptom burden, and reduced cost of care [23,40,41]. Neuropalliative care aligns with the foundational principles of oncology palliative care by prioritizing high-quality end-of-life care and facilitating the ability of terminally ill patients to die in their preferred setting, typically outside the hospital environment [42]. Since the 2018 expansion, the SHSC ALS clinic has grown from one palliative care physician to three. The clinic currently maintains a roster of approximately 250 patients per year who are followed longitudinally both in person and virtually—especially for those with mobility challenges or limited palliative support in the community. The establishment of a novel ALS-specific palliative care clinic will facilitate the rigorous evaluation of outcomes in the ALS population, including but not limited to hospital admission rates, goals-of-care documentation, and location of death. By enhancing our understanding of the nuanced and complex palliative care needs of patients and their families, this initiative aims to develop more responsive, patient-centered care strategies that address the unmet needs of this unique population.

### 5.2. Implications for Practice

As palliative care specialists gain experience managing ALS, prognostication and the timing of goals-of-care discussions may improve, supporting earlier and more effective integration of palliative care services. The findings of this study may help the PCCT optimize educational and organizational resources by informing clinician education on early identification of palliative care needs, prognostic uncertainty, and advance care planning, while supporting more structured referral pathways for patients with clinical decline or increasing care complexity.

Given the unpredictable and rapidly progressive nature of ALS, all clinical team members, including neurologists, clinical nurses, and allied health professionals, should engage in early, iterative conversations about ACP, patient values, and care preferences. Routine documentation of these discussions in the medical record ensures continuity across care settings and supports goal-concordant decision-making. ALS-specific evidence indicates that structured, team-based ACP engagement improves patient and caregiver satisfaction, clarifies future care expectations, and enhances alignment of care with patient goals [43].

### 5.3. Strengths

This study adds to the limited but growing body of research addressing the role of specialist palliative care in the ALS population. Our findings highlight significant challenges associated with late referrals to palliative care within SHSC, which can lead to poor health outcomes [44]. It highlights important questions for further investigation, including how the availability of specialized palliative care in hospitals shapes the delivery of care for ALS patients. There is a clear need for deeper exploration into the needs of patients and caregivers to ensure comprehensive, high-quality, and goal-concordant care.

### 5.4. Limitations

This study was limited by its inability to capture the involvement of community-based palliative care services, which likely play a significant role in the overall care of ALS patients. We were also unable to assess the impact of institutional initiatives, including the ACP/Goals-of-Care documentation tab, on advance care planning practices. Therefore, we could not determine whether implementation of this documentation tool influenced the frequency, timing, or completeness of advance care planning discussions or documentation. Additionally, due to limited resources, at the time of writing this manuscript, the most complete and accurate data available for analysis was seven years old. While the findings remain relevant today, changes to clinical practice and patient populations should be considered when interpreting the results. While the data captured some elements of healthcare utilization, it did not include clinical information such as symptom burden or intensity at the time of palliative care consultation. It also did not capture patient specific needs or outcomes, which can be indicators for measuring quality of care. For example, for the 22% of patients who were discharged home, there was no follow-up in our database regarding their end-of-life location, recurrent admissions or transitions in settings of care, which are pivotal junctures in an individual’s illness trajectory. Our data lacked details on personal goals and preferences associated with preferred location of death. In addition, this dataset only included new ALS patients, thus excluding those patients who may have been readmitted on a subsequent visit(s). This exclusion limits our ability to capture the full illness experience from time of diagnosis to end of life for ALS patients cared for at SHSC. Additionally, the study did not assess patient and family experiences related to specialist palliative care involvement. Data on patient and family satisfaction represent important quality indicators that should be addressed in future evaluations to improved understand the impact and quality of care provided for patients with ALS and their families [45].

## Figures and Tables

**Table 1:** Patient demographics captured in study.

**Age**	**n**	**%**
<65	13	15%
65–84	58	66%
85+	17	19%
Total	88	100%
**Gender**	**N**	**%**
Female	48	55%
Male	40	45%
Total	88	100%
**^1^ IP vs. OP**	**n**	**%**
Inpatient	85	97%
Outpatient	3	3%
Total	88	100%

^1^ IP = inpatient, OP = outpatient.

**Table 2:** Breakdown of the 42 patients who died in the hospital.

Time from Palliative Care Inpatient Consult to Death	*n* of Patients	*n* of Patients
Hours	4	10%
Days (<1 week)	27	64%
Weeks (1–4 weeks)	9	21%
<3 Months	2	5%
Total	42	100%

## Data Availability

Due to privacy and confidentiality requirements, the data are not publicly available. De-identified data may be made available from the corresponding author upon reasonable request and subject to institutional approval.

## Abbreviations

The following abbreviations are used in this manuscript:

LACP	Late Advance Care Planning
ALS	Amyotrophic Lateral Sclerosis
PCCT	Palliative Care Consult Team
SHSC	Sunnybrook Health Sciences Centre
PPS	Palliative Performance Scale
PCU	Palliative Care Unit
SPICT	Supportive and Palliative Care Indicators Tool
NECPAL	Necesidades Paliativas
DNR	Do not Resuscitate
ICU	Intensive Care Unit
ACP	Advance Care Planning
